# Machine learning based on nutritional assessment to predict adverse events in older inpatients with possible sarcopenia

**DOI:** 10.1007/s40520-024-02916-2

**Published:** 2025-02-22

**Authors:** Chengyu Liu, Hongyun Huang, Moxi Chen, Mingwei Zhu, Jianchun Yu

**Affiliations:** 1 Department of General Surgery, Peking Union Medical College Hospital, Chinese Academy of Medical Sciences and Peking Union Medical College, 1 Shuaifuyuan Road, Dongcheng District, Beijing, 100730 China; 2 Department of General Surgery, Department of Hepato-bilio-pancreatic Surgery, Beijing Hospital, National Center of Gerontology, Institute of Geriatric Medicine, Chinese Academy of Medical Sciences, 1 Dhua Road, Donghuamen Street, Dongcheng District, Beijing, 100730 China

**Keywords:** Machine learning, Aged, Possible sarcopenia, Adverse events, Prediction model, Nutritional assessment

## Abstract

**Background:**

The accuracy of current tools for predicting adverse events in older inpatients with possible sarcopenia is still insufficient to develop individualized nutrition-related management strategies. The objectives were to develop a machine learning model based on nutritional assessment for the prediction of all-cause death and infectious complications.

**Methods:**

A cohort of older patients with possible sarcopenia (divided into training group [70%] and validation group [30%]) from 30 hospitals in 14 major cities in China was retrospectively analyzed. Clinical characteristics, laboratory examination, Nutritional risk Screening-2002 (NRS-2002) and mini-nutritional Assessment-Short form (MNA-SF) were used to construct machine learning models to predict in-hospital adverse events, including all-cause mortality and infectious complications. The applied algorithms included decision tree, random forest, gradient boosting machine (GBM), LightGBM, extreme gradient boosting and neural network. Model performance was assessed according to learning a series of learning metrics including area under the receiver operating characteristic curve (AUC) and accuracy.

**Results:**

Among 3 999 participants (mean age 75.89 years [SD 7.14]; 1 805 [45.1%] were female), 373 (9.7%) had adverse events, including 62 (1.6%) of in-hospital death and 330 (8.5%) of infectious complications. The decision tree model showed a better AUC of 0.7072 (95% CI 0.6558–0.7586) in the validation cohort, using the five most important variables (i.e., mobility, reduced food intake, white blood cell count, upper arm circumference, and hypoalbuminemia).

**Conclusions:**

Machine learning prediction models are feasible and effective for identifying adverse events, and may be helpful to guide clinical nutrition decision-making in older inpatients with possible sarcopenia.

**Supplementary Information:**

The online version contains supplementary material available at 10.1007/s40520-024-02916-2.

## Introduction

According to the 2019 Asian Working Group on Sarcopenia consensus, sarcopenia is defined as age-related loss of muscle mass, combined with low muscle strength and/or low physical performance, which can be divided into three categories: possible sarcopenia, sarcopenia, and severe sarcopenia [1]. In a population-based longitudinal study, the prevalence of possible sarcopenia was estimated to be 46.0% among Chinese community-dwelling older adults (≥ 60 years) [2]. A meta-analysis of 15 studies (4014 patients) showed that 37% of older inpatients had sarcopenia [3].

Sarcopenia not only significantly increases the risk of falls and all-cause mortality of elderly populations, but also increases the risk of multiple postoperative complications in patients with digestive system tumors and other diseases, and prolongs the length of hospital stay of inpatients [4]. Timely intervention of patients with possible sarcopenia can prevent the further development of sarcopenia and powerfully improve the quality of life of individuals [5]. Nutritional intervention can improve muscle mass and functional status in older patients with sarcopenia [6–8].

Machine learning algorithms can process complex data to develop clinically useful tools, which can aid in accurate diagnosis and guide interventions in clinical practice [9]. Current studies have used machine learning to develop predictive models for adverse drug events, adverse cardiovascular and cerebrovascular events, 90-day all-cause mortality, and emergency department visits in older patients [10–12]. A recent study found that a sarcopenia score based on three variables (age, handgrip strength, and calf circumference) could be used for cardiovascular risk stratification in patients with abdominal obesity, and the combination of high sarcopenia score and low body mass index (BMI) can predict a higher incidence of composite adverse events including cardiovascular death in patients with abdominal obesity [13]. However, some predictors such as left ventricular ejection fraction and skeletal muscle area measured by computed tomography are not available in primary care or standard hospital settings [12, 14]. There is a lack of prognostic tools to predict adverse events in older inpatients with possible sarcopenia, which is insufficient to develop individualized nutrition-related management strategies. The objectives of our study were to develop a machine learning model based on nutritional assessment for the prediction of all-cause death and infectious complications in older inpatients with possible sarcopenia.

## Methods

### Study participants

This retrospective analysis from a multicenter nutritional survey of consecutively admitted older adult inpatients at 14 hospitals in China from March to May 2012. The original study was approved by the Ethics Committee of Beijing Hospital (registration number: LLKYPJ2012002A). Inclusion criteria: (1) age ≥ 65 years old; (2) inpatients with possible sarcopenia [Have a combination of low calf circumference and handgrip strength, calf circumference < 34 cm (male) or < 33 cm (female), handgrip strength < 28 kg (male) or < 18 kg (female)]; (3) no emergency surgery; (4) be willing to receive nutritional assessment and sign informed consent. Exclusion criteria: (1) emergency patients; (2) missing information of calf circumference or handgrip strength.

### Data collection

Demographic information such as age, gender, ethnicity, marital status, education level, medical insurance status, anthropometric indicators such as height, weight, BMI, calf circumference (CC), upper arm circumference, handgrip strength, and laboratory indicators were collected. Laboratory indicators included red blood cell (RBC) count, white blood cell (WBC) count, lymphocyte count, hemoglobin, albumin, total protein, direct bilirubin (DBIL), total bilirubin (TBIL), alanine aminotransferase (ALT), triglyceride (TG), total cholesterol (TC), blood urea nitrogen (BUN), and serum creatinine (Cr), etc.

Nutritional status was assessed using Nutritional Risk Screening 2002 (NRS-2002) and Mini-Nutritional Assessment Short Form (MNA-SF). The NRS-2002 was graded and scored according to the nutritional status, disease severity, and age, and a score of 3 or greater was considered nutritional risk [15]. MNA-SF, included the following six questions [16, 17]: (1) food intake reducing in the past 3 months because of the loss of appetite, digestive problems, chewing and swallowing difficulties; (2) weight loss in the past 3 months; (3) mobility; (4) whether the patient suffered from psychological stress or acute disease in the past 3 months; (5) neuropsychological problems; (6) BMI or calf circumference. Each question was scored 0–2 or 0–3 points, and according to the total score, the patients were divided into well-nourished (12–14 points), malnutrition risk (8–11 points), and malnourished (0–7 points).

Adverse events were defined as in-hospital death or infectious complications occurred during hospitalization. Infectious complications were defined as sterile tissue of pathogens and confirmed by the culture, or clinical signs and symptoms associated with infection and radiology or evidence of hematology.

### Data preprocessing and feature selection

Features with more than 30% missing values were excluded to minimize bias due to limited information. There were 35 features in total for predictors, and the number of missing data is shown in Supplementary Table 1. Multiple imputation is a commonly recommended approach to handle missing data during model development, with due consideration given to the uncertainty of missing data [18]. For the original data set, missing data were assumed to be missing at random and imputed five times through multiple imputation in the package “mice”. The imputation model included all predictor features and outcome variables, and then the imputed data was used to develop machine learning models.

The results of the machine learning algorithm were used for feature selection, and multivariate logistic regression was first used to select the features significantly associated with adverse events. Then, we used SHAP values (Shapley Additive exPlanations) to assist feature selection, and the model with the better predictive ability during feature dimension reduction was selected for further analysis. Finally, the predictors included in the best machine learning model selected were the most important features.

### Model development and comparison

From the derivation cohort, 70% of the participants was randomly selected for model construction (training group) and 30% for validation (internal validation group). A total of 35 characteristics was used to develop the prediction model. We selected six machine learning algorithms widely used in recent high-quality studies to develop the model to predict adverse events in older inpatients with possible sarcopenia, including decision trees, random forests (RF), gradient boosting machine (GBM), lightGBM, extreme gradient boosting (XGboost), and neural networks [19–22]. These R package “party”, “randomForest”, “gbm”, “lightgbm”, “xgboost” and “neuralnet” were used to run the above machine learning algorithms, respectively.

In the internal validation group, a series of commonly used machine learning evaluation indicators such as area under the receiver operating characteristic (ROC) curve (AUC), sensitivity, specificity, accuracy, and recall were used to evaluate the reliability of these models. The AUC was calculated by implementation in the R package “pROC”.

### Statistical analysis

Data analyses were conducted using R version 4.3.3. Continuous variables with normal distribution were presented as mean and standard deviation and compared using the t-test. Continuous variables with skewed distributions are presented as medians and interquartile ranges and compared with the use of the Mann-Whitney U test or the Kruskal-Wallis H test. Categorical variables were presented as numbers and percentages and compared using the chi-square test or Fisher’s exact test. AUC was used to evaluate its predictive ability and compared with Delong’s test. Two-sided *P* < 0.05 was considered statistically significant.

## Results

### Patient characteristics

**Fig. 1:**
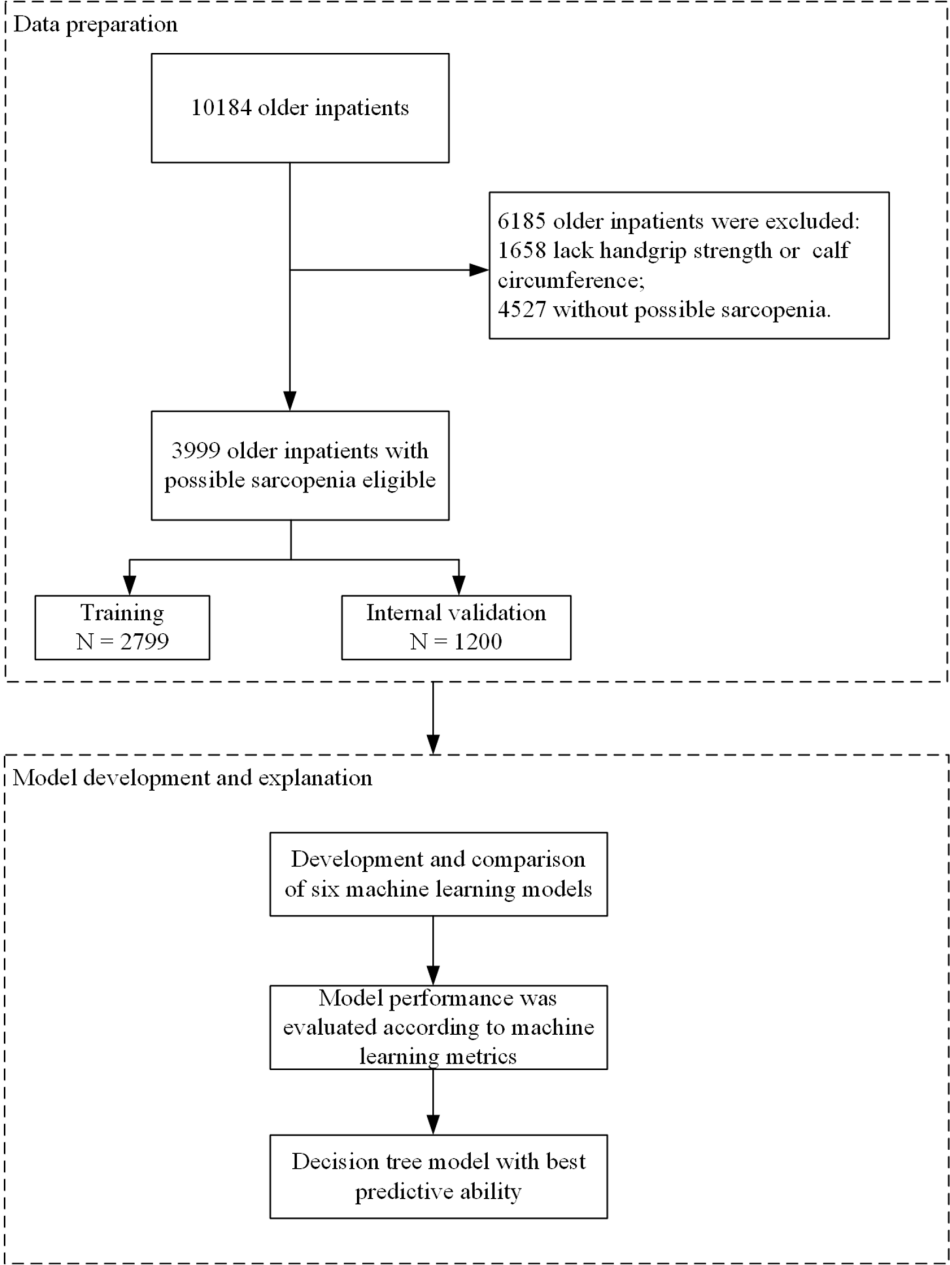
Flow chart of the study design

This retrospective study included 3 999 older inpatients with possible sarcopenia in the derivation cohort to develop predictive models. A total of 10 184 older patients from 30 hospitals in 14 major cities were investigated during the study period. 1 658 patients with lack of handgrip strength or calf circumference and 4 527 patients without possible sarcopenia were excluded. The 3 999 older inpatients with possible sarcopenia were assigned to a separate training set (70%) and an internal validation set (30%). Comparisons of demographic and clinical variables in included patients are shown in Table 1. Details of the study design are provided in Fig. 1.

**Table 1:** Baseline characteristics of 3999 older inpatients with possible Sarcopenia

Characteristic	Overall (*n* = 3999)	Patients without adverse events (*n* = 3607)	Patients with adverse events (*n* = 392)	*P* value
Gender female (%)	1805 (45.1)	1652 (45.8)	153 (39.0)	0.012
Age (years)	76.00 [70.00, 81.00]	75.00 [70.00, 81.00]	77.00 [72.00, 82.00]	< 0.001
Height (cm)	162.17 (8.13)	162.00 (8.09)	163.68 (8.36)	< 0.001
Weight (kg)	57.02 (10.17)	56.98 (10.11)	57.41 (10.73)	0.428
BMI (kg/m2)	21.50 [19.40, 23.70]	21.50 [19.50, 23.70]	21.30 [18.88, 23.70]	0.12
Education level (%)				0.048
Missing school	487 (12.2)	437 (12.1)	50 (12.8)	
Primary school	1262 (31.6)	1116 (30.9)	146 (37.2)	
Middle school	1466 (36.7)	1342 (37.2)	124 (31.6)	
College and above	784 (19.6)	712 (19.7)	72 (18.4)	
WBC count (10^9L)	6.93 (4.67)	6.79 (4.36)	8.24 (6.72)	< 0.001
RBC count (10^12L)	4.15 (5.08)	4.18 (5.34)	3.86 (0.73)	0.239
Hemoglobin (g/L)	119.59 (21.33)	119.88 (21.19)	116.98 (22.42)	0.011
Neutrophil count (10^9/L)	5.30 (7.19)	5.11 (6.86)	7.03 (9.51)	< 0.001
Lymphocyte count (10^9/L)	1.61 (2.92)	1.61 (2.93)	1.58 (2.87)	0.869
Albumin (g/L)	36.50 (6.31)	36.68 (6.32)	34.92 (6.05)	< 0.001
Hypoalbuminemia (%)	1401 (35.0)	1214 (33.7)	187 (47.7)	< 0.001
Total protein (g/L)	64.05 (8.20)	64.14 (8.13)	63.26 (8.84)	0.044
DBIL (umol/L)	6.64 (19.21)	6.76 (19.87)	5.60 (11.34)	0.259
TBIL (umol/L)	15.02 (26.51)	15.21 (27.44)	13.31 (15.44)	0.177
ALT (u/L)	24.87 (46.83)	24.99 (47.13)	23.80 (44.03)	0.633
TG (mmol/L)	1.34 (1.09)	1.34 (1.08)	1.34 (1.16)	0.996
TC (mmol/L)	4.29 (1.65)	4.31 (1.68)	4.13 (1.27)	0.036
BUN (mmol/L)	7.42 (11.79)	7.35 (11.97)	8.04 (10.04)	0.274
Cr (umol/L)	84.96 (65.16)	83.84 (61.52)	95.25 (91.56)	0.001
Handgrip strength (kg)	8.43 (7.68)	8.61 (7.68)	6.83 (7.50)	< 0.001
Calf circumference (cm)	29.74 (2.74)	29.78 (2.72)	29.35 (2.87)	0.003
Upper circumference (cm)	24.06 (3.37)	24.06 (3.32)	24.08 (3.75)	0.918
NRS-2002 grade	3.00 [2.00, 4.00]	3.00 [2.00, 4.00]	4.00 [2.00, 5.00]	< 0.001
Nutritional risk (%)	2337 (58.4)	2060 (57.1)	277 (70.7)	< 0.001
MNA-SF grade	11.00 [8.00, 12.00]	11.00 [9.00, 13.00]	9.00 [7.00, 12.00]	< 0.001
MNA-SF group (%)				< 0.001
well-nourished	1531 (38.3)	1431 (39.7)	100 (25.5)	
Malnutrition risk	1750 (43.8)	1582 (43.9)	168 (42.9)	
Malnourished	718 (18.0)	594 (16.5)	124 (31.6)	

All values are expressed as mean ± SD, median [IQR] or number (%); BMI, body mass index; WBC, white blood cell; RBC, Red Blood Cell; DBIL, Direct bilirubin; TBIL, Total bilirubin; ALT, Alanine aminotransferase; TG, triglyceride; TC, total cholesterol; BUN, Blood urea nitrogen; Cr, serum creatinine; NRS-2002, Nutritional Risk Screening 2002; MNA-SF, Mini-Nutritional Assessment Short Form

In the derivation cohort, 392 patients (9.8%) had adverse events, in-hospital death occurred in 79 (2.0%) patients, and 332 (8.3%) had infectious complications, including 249 (6.2%) pneumonia, 58 (1.5%) urinary tract infection, 18 (0.5%) abdominal infection, and 44 (1.1%) other infectious complications.

### Evaluation of model performance

**Fig. 2:**
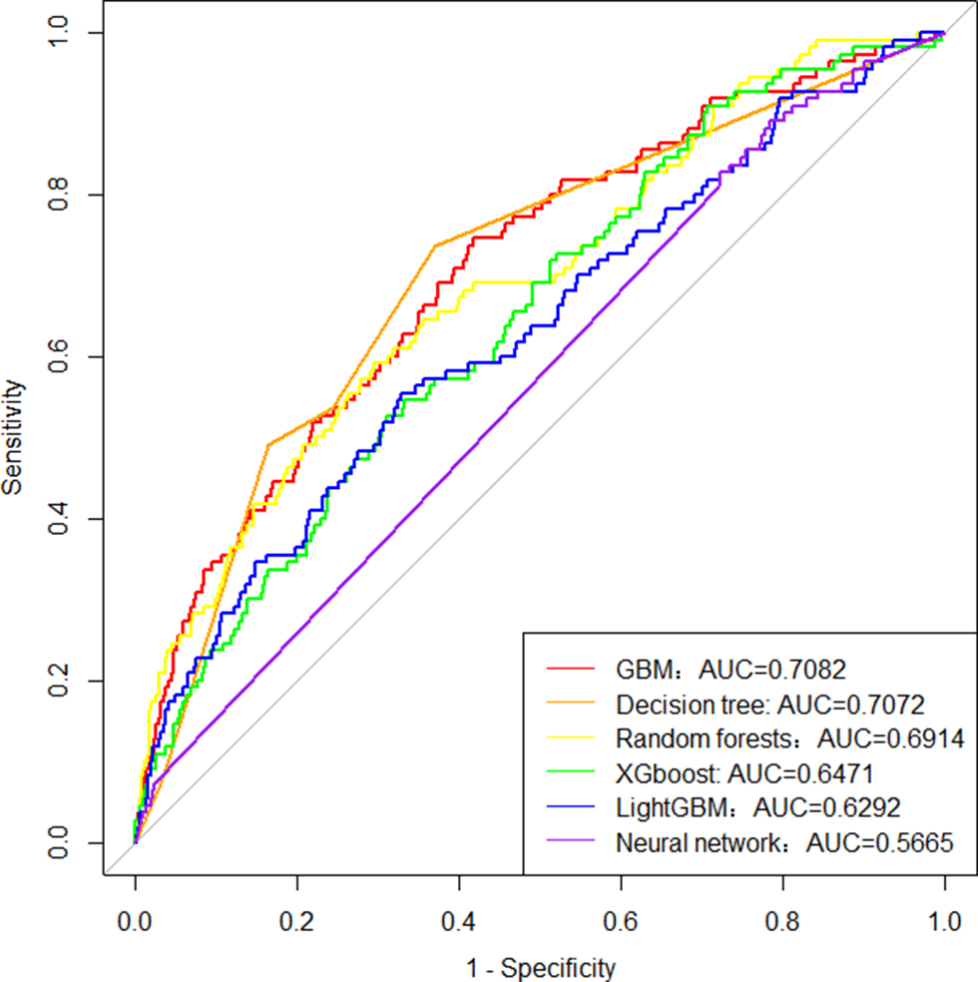
Receiver operating characteristic curve analysis of six machine learning models to predict adverse event in older inpatients with possible sarcopenia. AUC, area under the receiver operating characteristic curve; GBM, gradient boosting machine; XGboost, extreme gradient boosting

The data collected within 48 h after admission were used to develop machine learning models to predict adverse events. Among the six models, GBM, decision tree and RF models (AUC = 0.7082, 0.7072, 0.6914) had better prediction effect on adverse events. The prediction performance of XGboost, LightGBM and Neural network were not as good as those of the first three models (AUC = 0.6471, 0.6292, 0.5665), and the ROC curves were shown in Fig. 2.

There was no significant difference in the AUC among GBM, Decision tree and RF model (GBM vs. Decision tree, Z = 0.0470, *P* = 0.9625; GBM vs. RF, Z = 0.8903, *P* = 0.3733; Decision tree vs. RF, Z = 0.5768, *P* = 0.5641), and the first three models all had significantly higher predictive performance than XGboost (Z = 2.7283, 2.1064, 2.0851, *P* = 0.0064, 0.0352, 0.0371). The Accuracy, Sensitivity, Specificity, and Recall values of the model evaluation indicators of decision tree were all greater than 0.6, which was the best performance among the six machine learning models (Table 2).

**Table 2:** Comparison of prediction performance among prediction model

Items	AUC (95% CI)	Accuracy (95% CI)	Sensitivity	Specificity	Recall
GBM	0.7082(0.6561–0.7603)	0.5958(0.5674, 0.6238)	0.7454	0.5807	0.7454
Decision tree	0.7072(0.6558–0.7586)	0.64(0.6121, 0.6672)	0.73636	0.6302	0.7363
RF	0.6914(0.6379–0.7449)	0.695(0.6681, 0.721)	0.5818	0.7064	0.5818
XGboost	0.6471(0.5945–0.6996)	0.6742 (0.6468, 0.7006)	0.5181	0.6899	0.5181
LightGBM	0.6292(0.5716–0.6869)	0.6592(0.6316, 0.686)	0.5545	0.6697	0.5545
Neural network	0.5665(0.5235–0.6096)	0.2533(0.2289, 0.2789)	0.9090	0.1871	0.9090

AUC, area under the receiver operating characteristic curve; CI, confidence interval; GBM, gradient boosting machine; RF, random forest; XGboost, extreme gradient boosting

### Identification of important risk factors contributing to the model

**Fig. 3:**
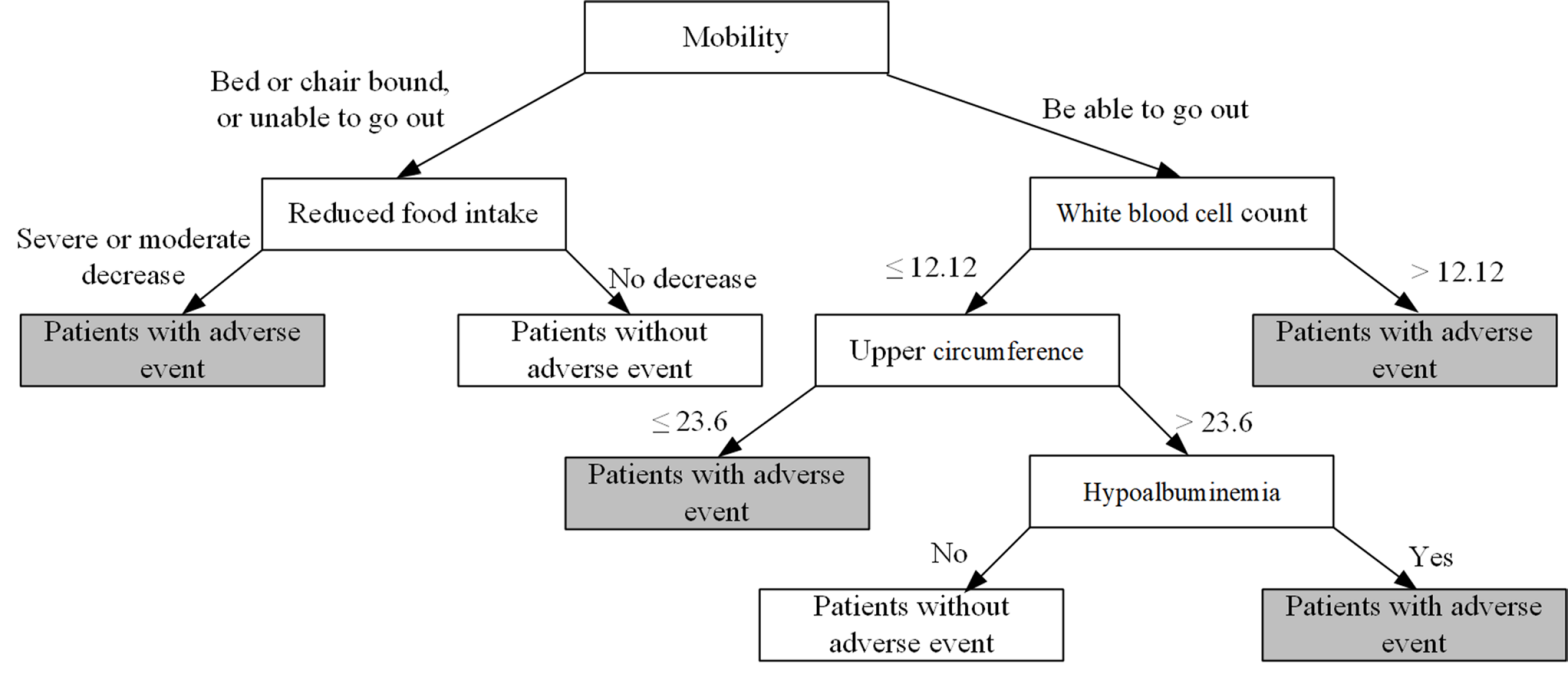
Visualization of decision tree models for predicting adverse events

The decision tree model used five variables: mobility, reduced food intake, white-cell count, upper arm circumference, and hypoalbuminemia (Fig. 3). Mobility, reduced food intake, WBC count, and hypoalbuminemia were positively associated with the risk of adverse events. On the contrary, the upper arm circumference was negatively correlated with the occurrence of adverse events.

## Discussion

In this study, we explored and compared six machine learning algorithms for the prognostic analysis of older inpatients with possible sarcopenia, and constructed a decision tree model to predict adverse events based on nutritional assessment and laboratory indicators. The decision tree model showed good predictive performance and may help early detection of high-risk adverse events in older inpatients with possible sarcopenia.

The present studies focused on the application of machine learning algorithms in disease diagnosis of sarcopenia or possible sarcopenia [23–27]. Based on the health and nutrition survey data of 8,092 participants, Korean scholars applied XGboost method to construct a model for predicting sarcopenia based on ocular omics data (AUC > 0.7) [23]. Liao et al. [24] included 242 patient data and developed a simple sarcopenia identification tool with a voting classifier in maintenance hemodialysis patients, which performed well in detecting possible sarcopenia. One sarcopenia model was developed using the baseline data of 4057 participants, and the external validation of 553 participants showed that the wide and deep model had good diagnostic performance for sarcopenia (external validation, AUC = 0.970, accuracy = 0.911) [27]. A recent study has found that LightGBM model including BMI, weight, waist circumference, and other physical characteristics and activities of related factors can effectively predict sarcopenia [28].

It has been demonstrated that machine learning models have accurate discrimination ability in the prediction of adverse events such as all-cause mortality and survival rate in diseases such as acute coronary syndrome, malignant tumor or surgery patients, which may help to guide clinical decision-making [19, 29–31]. Currently, there are no predictive tools for adverse events in older inpatients with possible sarcopenia, and we successfully developed a prediction model.

Malnutrition in older inpatients generally increases the risk of death and is associated with decreased activities of daily living during hospitalization and after discharge, but the main reason for the differences in the impact on clinical outcomes may be the differences in the nutritional assessment methods used [32]. The decision tree model used WBC count, upper arm circumference, and hypoalbuminemia to predict adverse events, in addition to commonly used indicators of nutritional assessment: decreased mobility and reduced food intake [33]. A longitudinal study involving 160,117 older women suggests that high WBC count was positively associated with total and coronary heart disease mortality, but less so with cancer mortality [34]. A cohort study of 19,575 participants demonstrated that mid-upper arm circumference was negatively and linearly associated with total and cardiovascular disease mortality [35]. Hypoalbuminemia has also been found to be associated with the risk of death and complications for a variety of diseases [36–38].

This study applied machine learning methods to predict adverse events in older inpatients with possible sarcopenia. The strength was that the constructed model enables clinicians to more quickly identify high-risk patients at risk for in-hospital adverse event, among other risks, who may benefit from treatment measures such as nutritional interventions. This study has several limitations. To begin with, this study was limited by the retrospective character and the previous design. There was no observational data on functional status, muscle mass measured by imaging, etc., which may lead to reduced predictive power of the prediction model. In addition, despite the use of multiple imputation for missing data, study bias may have occurred, with the possibility of missing certain important predictor variables and misestimating the relationship between predictors and outcomes. Furth more, our study lacked external validation and prospective validation. It is not possible to generalize the prediction model we constructed to a new environment at present, but external validation studies should be separated from the original model development [39].

Machine learning models such as neural networks may perform poorly on internal validation data due to overfitting. Moreover, only 35 features were included in our study, which may not meet the conditions that neural networks usually need a large number of parameters for training. Future studies that prospectively collect more features to construct prediction models may improve the prediction performance. Future directions include testing our model in external cohorts with similar nutritional assessments and measures of muscle mass, as well as extending adverse-event prediction models with the addition of radiographic correlates. Prospective studies identify high-risk patients and select targeted interventions to determine the role of the model in intervention guidance.

In conclusion, we successfully developed a machine learning model to predict in-hospital adverse events in older inpatients with possible sarcopenia based on clinical data such as nutritional assessment, anthropometric indicators, and laboratory indicators. The final decision tree model had better predictive performance for adverse events in the internal validation. According to the prediction model, individualized nutritional support, timely treatment, to give more attention, in order to improve the prognosis of older inpatients with possible sarcopenia.

## Electronic supplementary material

Below is the link to the electronic supplementary material.

Supplementary Material 1

## Data Availability

No datasets were generated or analysed during the current study.
