# Supplementary material for: Machine learning based on nutritional assessment to predict adverse events in older inpatients with possible sarcopenia
Source: Aging Clin Exp Res. 2025 Feb 22;37(1):48. doi: 10.1007/s40520-024-02916-2 (PMC11846711; doi:10.1007/s40520-024-02916-2)
Supplement: Supplementary file 1 — Supplementary Material 1 [file 40520_2024_2916_MOESM1_ESM.docx]

Supplementary table 1. Description of missing data

| Feature | Number of missing data | Complete rate |
| --- | --- | --- |
| Gender | 0 | 1 |
| Age | 0 | 1 |
| Nation | 145 | 0.964 |
| Height | 351 | 0.912 |
| Weight | 351 | 0.912 |
| BMI | 397 | 0.901 |
| Education level | 992 | 0.752 |
| Medical insurance | 0 | 1 |
| NRS-2002 weight loss | 63 | 0.984 |
| NRS-2002 decreased food intake | 30 | 0.992 |
| NRS-2002 grade | 3 | 0.999 |
| MNA-SF decreased food intake | 17 | 0.996 |
| MNA-SF weight loss | 23 | 0.994 |
| MNA-SF mobility | 46 | 0.988 |
| MNA-SF grade | 2 | 0.999 |
| Albumin | 242 | 0.939 |
| Hypoalbuminemia | 242 | 0.939 |
| Total protein | 255 | 0.936 |
| DBIL | 254 | 0.936 |
| TBIL | 221 | 0.945 |
| ALT | 193 | 0.952 |
| TG | 956 | 0.761 |
| TC | 1101 | 0.725 |
| BUN | 222 | 0.944 |
| Cr | 199 | 0.950 |
| WBC count | 146 | 0.963 |
| RBC count | 156 | 0.961 |
| Hemoglobin | 163 | 0.959 |
| Neutrophil count | 165 | 0.959 |
| Lymphocyte count | 158 | 0.960 |
| Handgrip strength | 0 | 1 |
| Calf circumference | 0 | 1 |
| Upper circumference | 64 | 0.984 |
| Nutritional risk | 3 | 0.999 |
| MNA-SF group | 2 | 0.999 |
| In-hospital death | 160 | 0.960 |
| Infectious complications | 139 | 0.965 |
| Adverse events | 139 | 0.965 |

BMI, body mass index; WBC, white blood cell; RBC, Red Blood Cell; DBIL, Direct bilirubin; TBIL, Total bilirubin; ALT, Alanine aminotransferase; TG, triglyceride; TC, total cholesterol; BUN, Blood urea nitrogen; Cr, serum creatinine; NRS-2002, Nutritional Risk Screening 2002; MNA-SF, Mini-Nutritional Assessment Short Form.
